# Risk Factors and Predictors of Outcomes in Hypoxic-Ischemic Encephalopathy in Neonates

**DOI:** 10.7759/cureus.73407

**Published:** 2024-11-10

**Authors:** Ruhi Shaligram, Balakrushna P Garud, Sudhir Malwade, Shailaja V Mane, Jasleen Dua, Mridu Bahal, Mrinali Thakur

**Affiliations:** 1 Pediatrics, Dr. D.Y. Patil Medical College, Hospital and Research Centre, Dr. D.Y. Patil Vidyapeeth (Deemed to be University), Pune, IND

**Keywords:** hypoxic-ischemic encephalopathy, intrauterine asphyxia, neurological deficits, perinatal hypoxia, risk factors

## Abstract

Background

Hypoxic-ischemic encephalopathy (HIE) in neonates results from oxygen deprivation at birth, often leading to long-term neurological issues like cerebral palsy. Early detection is key to improving outcomes, but HIE remains a significant cause of neonatal complications. Here we aim to study the risk factors and predictors of outcome in moderate to severe HIE in inborn Term babies in the neonatal intensive care unit (NICU)

Methods

This prospective observational study was conducted in the NICU at a tertiary care center between August 2022 and July 2024. Forty term neonates diagnosed with moderate to severe HIE based on clinical and MRI findings were included. The study recorded antepartum, intrapartum, and postnatal risk factors, and neonatal outcomes were followed up.

Results

In this study of 40 term neonates with HIE, 27 (67.5%) were male, 30 (75%) had a birth weight <2.5 kg, and 27 (67.5%) were delivered by cesarean section. Socioeconomic analysis revealed that 17 (42.5%) were from poor backgrounds. Of the mothers, 12 (30%) were over 30 years old and 19 (47.5%) used medications. Intrapartum factors included oligohydramnios in 13 (32.5%) and pregnancy-induced hypertension in 10 (25%). Postnatally, 28 (70%) required resuscitation, and 32 (80%) had a cord blood pH ≤7.0. MRI patterns showed 18 (45%) with basal ganglia/thalamus involvement and 22 (55%) with watershed lesions. At discharge, 39 (97.5%) were sent home, with 5 (12.5%) needing anti-seizure medications. No significant associations were found between MRI patterns and muscle tone or seizure activity.

Conclusion

This study highlights the complex interplay of maternal, intrapartum, and neonatal factors in the development of HIE. Identifying early risk factors is crucial for developing preventive and therapeutic strategies to reduce the burden of HIE-associated disabilities.

## Introduction

Hypoxic-ischemic encephalopathy (HIE) is a critical condition in neonatal medicine due to its potential to cause long-term neurological deficits. It significantly contributes to both morbidity and mortality, particularly in infants [1,2]. HIE results from brain damage caused by a hypoxic-ischemic event during the prenatal, intrapartum, or postnatal period, leading to restricted blood flow to the infant's brain [3]. The diagnosis of brain injury in infants who experience partial asphyxia at birth can be challenging, making it difficult for neonatologists and obstetricians to identify the condition.

HIE is among the most severe birth complications affecting full-term neonates [4]. In developed countries, it is estimated to occur in one to eight per 1000 live births, while in developing countries, the incidence can be as high as 26 per 1000 [5]. Depending on the severity of HIE, approximately 20% to 30% of affected infants die during the neonatal period [6]. Of the survivors, 33% to 50% suffer from permanent neurodevelopmental issues such as cerebral palsy (CP), reduced IQ, and cognitive impairments. Up to 60% of infants with HIE will either succumb or develop severe disabilities, including CP, epilepsy, and intellectual disabilities, by the age of two [7].

In term infants, the primary cause of hypoxic damage is intrauterine asphyxia due to circulatory complications, such as blood clots in placental arteries, placental abruption, or inflammatory processes [8]. These issues lead to prenatal depression, resulting in impaired oxygen and carbon dioxide exchange and severe lactic acidosis [9].

Given the severity of HIE, it is crucial for healthcare providers to prioritize identifying fetuses and newborns at risk for cerebral hypoxia-ischemia [10]. The incidence of HIE may be underestimated, and a deeper understanding of risk factors could be essential in developing new or adjunct treatments to reduce the burden of disability in neonates affected by HIE [11]. This highlights the importance of studying and identifying early risk factors and their correlation with short-term outcomes in HIE. Therefore, the aim of this study is to identify early risk factors and predictors of short-term outcomes in term infants with HIE.

## Materials and methods

Study design

The study was a prospective observational study conducted in the Neonatal Intensive Care Unit (NICU) Level III at Dr. D.Y. Patil Medical College, Hospital and Research Center, Pimpri, Pune. Newborns enrolled in the study were subsequently followed up at the "High-Risk OPD" of the same institute. Ethical committee clearance was obtained prior to the initiation of the study, and informed consent was acquired from the parents of all enrolled cases. The study period extended from August 2022 to July 2024.

Sample size calculation

The study considered a short-term outcome of seizures among moderate to severe HIE cases, estimated at 88.5%. With a confidence level of 95% and an acceptable attrition rate of 10%, the required sample size was calculated to be 40. The WINPEPI version 11.38 software (J.H. Abramson, Brixton Health, London, UK) was utilized for this calculation. A total of 40 term neonates were screened to study the risk factors and predictors of outcomes in HIE.

Patient selection criteria

All inborn term babies admitted to the NICU with perinatal hypoxia and diagnosed with HIE based on MRI scans performed between days 5 and 7 of life were screened for participation. Only those diagnosed with moderate to severe HIE were included in the study. Exclusion criteria involved syndromic term babies, those with inborn metabolic errors, congenital anomalies, cases of mild HIE, and those unwilling to participate.

Study variables

The predictive variables recorded for death or disability after HIE included antepartum-associated risk factors (e.g., maternal age, nationality, gravidity, unbooked maternal comorbidity like diabetes, preeclampsia/hypertension, urinary tract infection/vaginitis), intrapartum-associated risk factors (e.g., gestational age, malpresentation, meconium-stained amniotic fluid, premature rupture of membranes (PROM), abnormal cardiotocographic findings, labor augmentation, prolonged second stage of labor, placental abnormalities, mode of delivery, instrumental delivery, sentinel events), and neonate's data (e.g., gender, birth weight, and Apgar score). The hospital’s eligibility criteria for therapeutic hypothermia included pH ≤7, base deficit (BD) ≥16, and Apgar score ≤5 at 10 min.

Data collection

Data collection involved reviewing the newborns during their NICU stay and interviewing parents using a validated questionnaire. Necessary investigations, including neuroimaging and ophthalmic evaluation, were conducted.

Upon admission to the NICU, oxygen and ventilatory support were provided based on Downe's score. Intravenous fluids were administered at 2/3 maintenance with 10% dextrose, and patients were kept nil by mouth. Euglycemia and euthermia were maintained. The NICU followed developmental supportive care protocols, including maintaining noise levels below 65 decibels, nesting, and early sensory stimulation. Patients were monitored for oxygen saturation, heart rate, blood pressure, urine output, and neurological status.

Confirmation of the diagnosis of HIE was based on a combination of historical factors (e.g., umbilical cord complications, prolonged labor, fetal distress), clinical findings in the newborn (e.g., acidemia with pH <7.0, low Apgar scores, neurologic dysfunction, multiorgan involvement), and radiological evidence from a 3T MRI scan. Cases were categorized into Stages 2 and 3 using Sarnat & Sarnat staging. A 3T MRI scan was performed between days 5 and 7 of life for all cases. Discharge planning involved ensuring that patients were stable and on full oral feeds, with regular follow-up calls scheduled.

Statistical analysis

Data were entered and analyzed using the Statistical Package for Social Sciences (SPSS) for Windows, Version 28.0 (Armonk, NY: IBM Corp). Confidence intervals were set at 95%, and a p-value of ≤0.05 was considered statistically significant. Categorical variables were presented in frequency tables, while continuous variables were expressed as mean ± standard deviation. The chi-square test was applied to check for associations between variables.

## Results

In this study, the gender distribution among the 40 term neonates diagnosed with HIE showed that 13 (32.5%) were female and 27 (67.5%) were male. The socioeconomic status analysis of the study population showed that 17 neonates (42.5%) were from poor socioeconomic backgrounds, 21 neonates (52.5%) belonged to the lower middle class, and two neonates (5%) were from the upper middle class. 

Among the 40 neonates included in the study, 36 (90%) were born at term, while four (10%) were classified as late preterm. The birth weight distribution showed that 30 neonates (75%) had a birth weight of less than 2.5 kg, while 10 neonates (25%) had a birth weight of more than 2.5 kg. Regarding the mode of delivery, 27 (67.5%) neonates were delivered via lower segment cesarean section (LSCS), while 13 (32.5%) were delivered via normal vaginal delivery.

The distribution of neonates based on maternal factors is presented in Table 1. The maternal age distribution revealed that 30% (12) of the neonates had mothers above 30 years of age, and 70% (28) had mothers below 30 years. In terms of Gravida, 65% (26) of neonates’ mothers had experienced their first pregnancy, while 35% (14) had experienced more than one pregnancy. The presence of underlying disease was noted for 32.5% (13) mothers, while 67.5% (27) mothers had no underlying disease. The use of medicines was reported by 47.5% (19) mothers, whereas 52.5% (21) did not use any significant maternal medication.

**Table 1:** Distribution of neonates based on maternal factors

Variable	Subcategory	Number	Percentage
Maternal age (years)	>30	12	30
	<30	28	70
Gravida	1	26	65
	≥1	14	35
Underlying disease	Yes	13	32.5
	No	27	67.5
Maternal medicines	Yes	19	47.5
	No	21	52.5

The distribution of intrapartum factors among neonates was analyzed in this study (Table 2). Regarding plurality, 92.5% (37) involved single pregnancies, while twins accounted for only 7.5% (3). Meconium staining was observed in 27.5% (11) of the neonates, with the remaining 72.5% (29) showing no staining. Uteroplacental insufficiencies were identified in 12.5% (5) of cases, and placental abnormalities were noted in 5% (2). Oligohydramnios was reported in 32.5% (13) of the neonates. Pregnancy-induced hypertension (PIH) was found in 25% (10) of the neonates, while preeclampsia and eclampsia were each present in 5% (2). PROM occurred in 25% (10) of the neonates, with the remaining 75% (30) unaffected while 5% (2) were infants of diabetic mothers.

**Table 2:** Distribution of intrapartum factors among the neonates PIH, pregnancy-induced hypertension; PROM, premature rupture of membranes.

Variable	Subcategory	Number (n)	Percentage
Plurality	Single	37	92.5
	Twins	3	7.5
Meconium stain	Yes	11	27.5
	No	29	72.5
Uteroplacental insufficiencies	Yes	5	12.5
	No	35	87.5
Placental abnormalities	Yes	2	5
	No	38	95
Oligohydramnios	Yes	13	32.5
	No	27	67.5
PIH	Yes	10	25
	No	30	75
Preeclampsia	Yes	2	5
	No	38	95
Eclampsia	Yes	2	5
	No	38	95
PROM	Yes	10	25
	No	30	75
Infants of diabetic mother	Yes	2	5
	No	38	95

Fetal factors revealed that 10% (4) of the neonates had anemia, 15% (6) had infections, 2.5% (1) experienced bradycardia, and 5% (2) presented in breech position at birth. Meconium-stained liquor was present in 27.5% (11) of cases, while persistent pulmonary hypertension of the newborn (PPHN) was noted in 20% (8) of the participants (Table 3).

**Table 3:** Distribution of fetal factors among the neonates PPHN, persistent pulmonary hypertension of the newborn.

Variable	Subcategory	Number (n)	Percentage
Anemia	Yes	4	10
	No	36	90
Infection	Yes	6	15
	No	34	85
Bradycardia	Yes	1	2.5
	No	39	97.5
Breech presentation	Yes	2	5
	No	38	95
Meconium-stained liquor	Yes	11	27.5
	No	29	72.5
PPHN	Yes	32	20
	No	8	80

Postnatal factors showed that all 40 neonates had an Apgar score below 7 at both 1 and 5 min, but by 10 min, only 70% (28) still had a score below 7. Resuscitation was necessary for 70% (28) of the neonates, while 30% (12) did not require it. Cord blood analysis indicated that 80% (32) had a pH of 7 or lower, along with a base excess of 12 or more (Table 4).

**Table 4:** Distribution of postnatal factors among the neonates

Variable	Subcategory	Number (n)	Percentage
Apgar score at 1 min	<7	40	100
	≥7		
Apgar score at 5 min	<7	40	100
	≥7		
Apgar score at 10 min	<7	28	70
	≥7	12	30
Resuscitation	Yes	28	70
	No	12	30
Cord blood			
pH ≤7	Yes	32	80
	No	8	20
Base excess ≥12	Yes	32	80
	No	8	20

Ventilation types used among the patients showed that 50% (20) were on mechanical ventilators, 42.5% (17) were on high-flow nasal cannula or nasal continuous positive airway pressure, and 7.5% (3) were on room air. HIE staging based on Modified Sarnat and Sarnat Staging revealed that 85% (34) were in Stage 2, followed by 10% (4) in Stage 1, and 5% (2) were in Stage 3 (Table 5).

**Table 5:** HIE staging HIE, hypoxic-ischemic encephalopathy.

HIE staging (Modified Sarnat and Sarnat Staging)	Number	Percentage
1	4	10
2	34	85
3	2	5
Total	40	100

The analysis of MRI patterns revealed that 45% (18) had basal ganglia/thalamus (BGT) involvement, while 55% (22) had watershed patterns. Normal tone was displayed by 85% (34), while 15% (6) displayed abnormal tone. Normal reflexes were observed in 80% (32), whereas 20% (8) had abnormal reflexes. In terms of consciousness, 87.5% (35) had a normal level, and 12.5% (5) were hyperalert. Anti-seizure medication was required in 12.5% (5) of cases, and 2.5% (1) experienced feeding difficulties. Overall, 97.5% (39) of the neonates were discharged, with 2.5% (1) leaving against medical advice (Table 6).

**Table 6:** HIE outcome at the time of discharge HIE, hypoxic-ischemic encephalopathy; DAMA, discharged against medical advice.

Neurological variables	Subcategory	Number	Percentage
Tone	Normal	34	85
	Abnormal	6	15
Reflex	Normal	32	80
	Abnormal	8	20
Level of consciousness	Normal	35	87.5
	Hyperalert	5	12.5
Started anti-seizure medication	Yes	5	12.5
	No	35	87.5
Feeding difficulty	Yes	1	2.5
	No	39	97.5
HIE outcome	Discharged	39	97.5
	DAMA	1	2.5

The analysis of MRI patterns and HIE outcomes indicated that 35% (14) of the BGT group and 50% (20) of the watershed group exhibited normal muscle tone, while 10% (4) of the BGT group and 5% (2) of the watershed group showed abnormal muscle tone. However, no significant association was found between MRI patterns and muscle tone (p = 0.247) (Table 7).

**Table 7:** Association of MRI (brain) pattern with HIE outcome-muscle tone changes MRI, magnetic resonance imaging; HIE, hypoxic-ischemic encephalopathy.

MRI pattern	HIE outcome-muscle tone		Total	Percentage	Chi-square; p-value
	Normal	Abnormal			
Basal ganglia/thalamus	14	4	18	45	Chi value = 1.33; p-value = 0.247
Watershed pattern	20	2	22	55	
Total	34	6	40	100	

In terms of seizures, 25% (10) of infants in the BGT group and 35% (14) in the watershed group did not experience seizures, while 20% (8) in each group had seizures. However, the correlation between MRI patterns and seizure activity was not statistically significant (p = 0.604) (Table 8).

**Table 8:** Association of MRI pattern with seizures MRI, magnetic resonance imaging; CNS, central nervous system; BGT, basal ganglia/thalamus.

MRI pattern	CNS (seizure)		Total	Percentage	Chi-square; p-value
	Absent	Present			
BGT	10	8	18	45	Chi value = 0.26; p-value = 0.604
Watershed pattern	14	8	22	55	
Total	24	16	40	100	

All 40 participants were discharged, with no deaths reported.

## Discussion

HIE is the leading cause of neonatal encephalopathy and a significant contributor to neonatal morbidity and mortality worldwide, causing 814,000 deaths annually and ranking as the fifth leading cause of death in children under five. In this study, 40 neonates were included, of which 67.5% (27) were male and 32.5% (13) were female. In a similar study, Debillon et al. observed a similar 53.8% male prevalence [12]. The study by Peebles et al. included 37 infants who met HIE inclusion criteria. They had a female predominance, where 54.1% (20) were female and 46.0% (17) were male [13]. There is a possibility that the variation in HIE between males and females is connected to the fact that males and females have different levels of steroid hormones and different levels of motor function [14].

In this study, approximately one-third of the newborns (n = 30, 75%) had a birth weight of less than 2.5 kg. Low birth weight is frequently associated with neonatal hypoxia-ischemia. A significant increase in the incidence of moderate to severe HIE was observed among newborns with low birth weight. Birth weight below 3.0 kg or above 4.0 kg is recognized as a risk factor for moderate to severe neonatal HIE. Our findings suggest that low birth weight is an independent risk factor for the development of HIE. These results are consistent with several previous studies [15,16].

In our study, a higher proportion of neonates, 67.5% (n = 27), were delivered via LSCS, while 32.5% (n = 13) were born through vaginal delivery. These findings are consistent with a similar study conducted by Torbenson et al., which reported that 53.9% of HIE cases involved cesarean delivery following labor, 19.2% were delivered by cesarean without labor, 23.1% had spontaneous vaginal deliveries, and 3.8% were delivered via operative vaginal procedures [17].

The presence of underlying disease was noted in the mothers of 13 (32.5%) participants, while 27 mothers (67.5%) had no underlying disease. The use of medicines was reported by 19 (47.5%) participants’ mothers, whereas 21 (52.5%) did not use any maternal medication. In the study by Ezenwa et al., the mean maternal age was 28.02 years with a standard deviation of 5.33 years, and the median parity was 2, with a range of 1-3 [18].

In this study, meconium staining was noted in 11 (27.5%) participants, while 29 (72.5%) had none. In a study by Peebles et al., placental abruption, ruptured uterus, moderate-to-heavy meconium-stained amniotic fluid, and delivery by cesarean section were determined to be the independent intrapartum risk factors for HIE [13]. In this study, PROM occurred in the mothers of 10 (25%) participants, with 30 (75%) having no PROM. These findings are commensurate with those of Melaku et al., who reported that more than half of the cases (53.1%) and more than a third of the cases (34.9%) had a duration of more than 12 h in relation to the duration of labor and membrane rupture during the intrapartum period [19]. Uteroplacental insufficiencies were present in 5 (12.5%) cases, while placental abnormalities were found in 2 (5%) cases. Oligohydramnios was reported in 13 (32.5%) participants. PIH affected 10 (25%) participants, whereas preeclampsia and eclampsia were each present in 2 (5%) cases. A recent study by Mithra et al. found that the majority of neonates with HIE were born to first-time mothers, with 60% experiencing gestational hypertension, post-dated pregnancies, intrauterine growth restriction, anemia, and gestational diabetes. Meconium-stained fluids, respiratory distress, birth asphyxia, and sepsis were major neonatal morbidities. Doppler alterations were statistically associated with maternal complications, gestational period, neonatal morbidity, low APGAR at birth, and neonatal mortality [20]. Another study by Sarma et al. reported that the oligohydramnios had a higher incidence of newborns with HIE [21]. Zamzami et al. conducted a study to identify proximal risk factors associated with perinatal hypoxic encephalopathy and its short-term complications. They found that fetal acidosis, indicated by a cord pH ≤7, occurred in 31 cases (5.6%), while a BD ≥12 mmol/L was seen in 59 cases (10.7%). Intrapartum proximal risk factors included abnormal fetal heart rate patterns, prolonged labor, vacuum delivery, PIH, fetal growth restriction, and abruptio placenta [22].

Postnatal factors revealed that all 40 participants had an Apgar score of less than 7 at 1 and 5 min, but at 10 min, only 28 (70%) had a score of less than 7. Resuscitation was required for 28 (70%) participants, while 12 (30%) did not require it. Our study concurs with several previous studies done by Scheidegger et al., Chandrasekaran et al., and Shah et al. [10,23,24].

In our study, HIE staging based on Modified Sarnat and Sarnat Staging revealed that 34 (85%) were in Stage 2, followed by 4 (10%) in Stage 1, and 2 (5%) were in Stage 3. These results are consistent with the study by Scheidegger et al., who reported that Sarnat Stage 1 was seen in 16 (9.2%), Sarnat Stage 2 was seen in 126 (72.4%), Sarnat Stage 3 was seen in 32 (18.4.%) [10]. Kooij et al. observed that the corpus callosum was diminishing in 11 of 30 children (37%) with Sarnat Stage 1 HIE and in 19 of 36 (53%) with Stage 2 HIE, in contrast to eight of 49 controls (16%). The posterior portion of the corpus callosum was discovered to be smaller in size, which was associated with motor deficits [25].

In our study, 20 out of 40 participants (50%) had multiorgan dysfunction. Seizures were absent in 10 (25%) infants in the BGT group and 14 (35%) in the watershed group, while 8 (20%) in each group experienced seizures, with no significant correlation between MRI patterns and seizure activity. A study by Logitharajah et al. aimed at identifying the causes, brain injury patterns, and their predictive value in preterm infants with HIE found a seizure incidence of 82% (38/45) and multiorgan failure in 42% (21/50) of cases [26].

In our study, the analysis of MRI patterns revealed that 18 (45%) had BGT involvement, while 22 (55%) had watershed patterns. Neurological evaluation at discharge revealed that 34 (85%) patients had normal muscle tone, while 6 (15%) had abnormal tone. Additionally, 32 (80%) patients had normal reflexes, while 8 (20%) had aberrant reflexes. The level of consciousness was normal in 35 (87.5%) patients, while 5 (12.5%) were hyperalert. Brain injury patterns in preterm infants with indications of HIE have been assessed in only a limited number of studies. The association between MRI patterns and HIE outcomes in our study showed that 14 (35%) of the BGT group and 20 (50%) of the watershed group had normal muscle tone, while 4 (10%) of the BGT group and 2 (5%) of the watershed group exhibited abnormal muscle tone. However, this association was not statistically significant (p = 0.247). In a comparable investigation, Fortin et al. discovered that 140 (82.4%) of the 170 children with MRI findings indicative of hypoxic-ischemic injury had documented neonatal encephalopathy, while 29 (17.0%) did not [27].

Limitations

This study adds critical information to assess the complex interplay of maternal, intrapartum, and neonatal factors in the development of HIE. However, one limitation of this study is the relatively small sample size of 40 neonates, which may limit the generalizability of the findings and reduce statistical power. Additionally, the study relies on single-center data, which might introduce institutional biases and may not fully represent the broader population. Furthermore, the lack of randomization and control for potential confounding variables, such as variations in treatment protocols or maternal health conditions, could influence the results.

## Conclusions

This study underscores the complexity of HIE in term neonates, demonstrating that while a range of intrapartum and postnatal factors contribute to the condition, specific patterns of brain injury and their implications for neurological outcomes remain elusive. Despite identifying key associations and patterns, such as the predominance of BGT and watershed lesions, the study highlights the need for ongoing research to better understand the long-term impacts of these findings and to develop targeted strategies for prevention and treatment.
